# Impact of COVID-19 upon changes in emergency room visits with chest pain of possible cardiac origin

**DOI:** 10.1186/s13104-020-05381-y

**Published:** 2020-11-18

**Authors:** Adeel A. Butt, Anand Kartha, Nidal Asaad, Aftab M. Azad, Roberto Bertollini, Abdul-Badi Abou-Samra

**Affiliations:** 1 Department of Medicine, Hamad Medical Corporation, PO Box 3050, Doha, Qatar; 2 Weill Cornell Medical College, Doha, Qatar; 3 Weill Cornell Medical College, New York, NY USA; 4 Ministry of Public Health, Doha, Qatar

**Keywords:** COVID-19, Emergency Department, SARS-CoV-2, Qatar, Acute illness

## Abstract

**Objectives:**

A decrease in Emergency Department (ED) visits for cardiac conditions has recently been reported from the US and Western Europe due to the COVID-19 pandemic. The data are still scant, and the correlation between cardiac symptoms and confirmed diagnoses are not available. There are no reports on changes in ED volumes at a national level, or from countries in the Asia-Middle Eastern region. We report data from national referral centers for tertiary care and cardiac care centers in Qatar, which see > 80% of cardiac emergencies in the country.

**Results:**

We analyzed 102,033 ED visits in the COVID-19-era (March–April 2020 and 2019) and determined the proportion presenting for cardiac symptoms and their confirmed diagnoses. We observed a 16–37% decline in ED volumes overall, with a 25–50% decline in patients presenting with cardiac symptoms in March and April 2020 compared with March and April 2019. Among those presenting with cardiac symptoms, we observed a 24–43% decline in cardiac diagnoses in March and April 2020 compared with March and April 2019.

## Introduction

Earlier anecdotal reports of a decline in acute care hospitalizations during the current COVID-19 pandemic have been confirmed with recent publications reporting a decline in overall admissions and admission for acute coronary syndrome [1–6]. These reports have all been from the US or Western Europe with relative homogenous populations. Data from countries with large migrant worker and more diverse populations are not available. Changes in volumes and visits to the Emergency Department (ED) for cardiac symptoms, and their correlation with final diagnosis in the COVID-19-era compared with pre-COVID-19 era are also largely unknown. Our aim was to determine the change in volumes of patients presenting to large Emergency Departments with cardiac symptoms before and after the current COVID-19 pandemic, and to determine the proportion of a confirmed cardiac diagnosis among them.

## Main text

### Methods

In the State of Qatar, over 80% of patients with suspected acute coronary events are seen in two hospitals: Hamad General Hospital (HGH), which is the nation’s largest tertiary care referral center and the Heart Hospital (HH), which is the only specialty hospital in the nation designated specifically for cardiac care. Both hospitals are accredited by the Joint Commission International and utilize the same electronic health records system (Cerner^®^). Patients retain the same unique medical record number across both hospitals. The first COVID-19 patient in Qatar was diagnosed on February 29, 2020. We determined the number of individuals presenting to the EDs at the above mentioned hospitals during March and April 2020 (COVID-19-era) and compared it with the numbers in March and April 2019 (pre-COVID-19-era). Each month in the COVID-19 was era was compared with the same month in the prior year. At presentation, a trained nurse obtained the history of presenting complaint. Patients with acute chest pain with characteristics suggestive of cardiac origin (e.g. radiation to the left arm, other concomitant symptoms), shortness of breath (except when with cough and fever), palpitations and syncope/near syncope were categorized as having cardiac symptoms by three physicians among the authors (AAB, AK, AA), who independently reviewed all presenting complaints and assigned this category. Any disagreement was resolved by further review and mutual consensus.

### Results

A total of 102,033 ED visits were recorded over the study period. Compared with the same month in 2019, a 16.2% decline in total ED visits was noted in March 2020 and a 37.4% decline in April 2020 (Table 1). A similar decline in patients presenting with cardiac symptoms was also noted, with a 25.0% decline in March 2020 and a 49.9% decline in April 2020 compared with the same month in 2019. Among those presenting with cardiac symptoms, a sharp decline in confirmed cardiac diagnoses was also noted (24.3% in March 2020; 42.6% in April 2020). A list of all patients with their presenting symptoms, the breakdown of cardiac symptoms and confirmed diagnoses among those with cardiac symptoms are provided in Additional file 1: Tables S1–S3 respectively.

**Table 1:** Total Emergency Department visits for cardiac symptoms (chest pain likely cardiac, palpitations, shortness of breath and syncope/near syncope) and a breakdown of confirmed cardiac diagnoses among those with cardiac symptoms in months after and before COVID-19 cases

	2019–03	2019–04	2020–03	2020–04				
Total ED visits	29,908	28,950	25,049	18,126				
% change from previous year	–	–	− 16.2%	− 37.4%				

	N	%	N	%	N	%	N	%
Total ED visits for cardiac symptoms	3185	10.6	2941	10.2	2389	9.5	1472	8.1
% change from previous year	–	–	–	–	− 25.0%		− 49.9%	
Cardiac diagnoses among those with cardiac symptoms	445	14.0	350	11.9	337	14.1	201	13.7
% change from previous year	–	–	–	–	− 24.3%		− 42.6%	
Acute coronary syndrome	191	6.0	167	5.7	170	7.1	113	7.7
Angina or coronary artery disease without acute coronary syndrome	93	2.9	74	2.5	32	1.3	7	0.5
Arrythmia	78	2.4	42	1.4	47	2.0	26	1.8
Cardiac arrest	20	0.6	14	0.5	20	0.8	19	1.3
Congestive heart failure	62	1.9	47	1.6	63	2.6	35	2.4
Valvular disease	1	0.0	6	0.2	5	0.2	1	0.1

1. Denominator for percentage calculation is the total number of ED visits for that month

2. Denominator for percentage calculation is the total number of ED visits for cardiac symptoms

### Discussion

There are limited data regarding the changes in volumes of patients presenting to emergency department with acute cardiac diseases during the COVID-19 pandemic. To our knowledge, this is the first study to provide detailed information on cardiac symptoms and confirmed cardiac diagnoses in the COVID-19-era compared with pre-COVID-19-era. We observed a sharp decline in patients presenting to a specialty cardiac hospital with cardiac symptoms in the COVID-19-era.

We previously reported a decline in overall hospital admission rates and a decline in overall emergency department visits in tertiary care and specialty hospitals in the COVID-19 era [7, 8]. However, our previous paper lacked the reasons for presenting to the emergency department and final diagnoses for each visit, particularly regarding cardiac disease. In the current report, we demonstrate a 25–50% year-on-year decline in patients presenting with symptoms of cardiac origin. The number and proportion of persons with a confirmed cardiac diagnosis among those presenting with cardiac symptoms also dropped in the COVID-19 era. The reasons for these declines are most likely related to the travel and movement restrictions in the COVID-19-era, since all other parameters for patient access, flow and care remained unchanged [8]. These declines may be among patients with serious conditions who were otherwise unable to travel to an ED, or a self-conclusion by patients considering the symptoms to be non-emergent in nature. It is also possible that an excessive number of patients with symptoms remotely suggestive of an acute cardiac condition were presenting to the emergency departments in the pre-COVID-19 era even when they were not entirely convinced of the cardiac origin of symptoms. Finally, a perceived fear of acquiring COVID-19 infection from the hospital or during travel to or from the hospital may have been an additional factor contributing to this decline.

Restriction of movement and other public health measures can significantly impact the trajectory of an epidemic and can also affect the access and flow of patients seeking medical care. In Qatar, a host of public health measures were implemented gradually that promoted physical distancing, including closing retail stores in malls and shopping centers, closing entertainment and dining facilities, postponing or canceling large sports events and conferences, suspending classes in schools and universities, and mandating working from home for 80% of workers in the public and private sectors. These measures were gradually implemented in March 2020 and remained in place in April 2020. Whether these measures directly impacted the volumes and reasons for ED visits is currently unknown [9].

The sharp decline in overall ED visits confirms anecdotal reports and recently published reports from regional hospitals and healthcare systems in the US and Western Europe [2, 3, 6, 10]. These published reports indicate a decline in number of acute coronary syndrome cases, a reduction in out of hospital cardiac arrests, a reduction in imaging for acute stroke and a reduction in cardiac catheterization laboratory activation for patients with ST-segment elevation. These trends are worrisome since acute cardiac conditions generally require immediate intervention and appropriate emergency and/or inpatient care for achieving optimal outcomes. Regardless of the cause of these sharp declines, a surge of patients may be expected with relaxation of restriction of movement and appropriate steps need to be taken in anticipation of such a surge. It is also critical to determine any increase in cardiac related mortality in the community and institute interventions to mitigate such risk.

### Conclusions

In conclusion, a sharp decline in total ED visits, as well as visits for cardiac symptoms was observed at a national level, with a parallel decrease in number and proportion of persons with a confirmed cardiac diagnosis. A post-COVID-19 surge in patients with these conditions may be anticipated and preparations should be made to address it.

## Limitations

Data were collected as part of routine clinical care and not specifically collected for clinical research. However, there was uniformity in collection of data since all clinical staff were required to follow uniform clinical protocols to assess patients. The cardiac diagnoses were those made in the emergency department. It is possible that new diagnoses may have developed during the hospital stay or the final emergency department diagnoses may have been modified after further testing and evaluation. In the future, a more structured evaluation of patients with known or suspected COVID-19 infection who present to the emergency departments should be undertaken to understand its true impact. It is also important to determine the volumes and outcomes of patients with cardiac symptoms and diagnoses after the COVID-19 related restrictions are lifted to determine the population level impact of these changes. Other well defined manifestations of cardiac disease include myocarditis and thromboembolic events which were not diagnosed in the emergency department in our study. It will be important to study the short-terms and long-term incidence/prevalence of these conditions in patients presenting to the emergency departments with COVID-19 and cardiac symptoms.

## Supplementary information

**Additional file 1: Table S1.** Chief complaints of patients presenting to the Emergency Departments at all participating hospitals over the study period (listed alphabetically). **Table S2.** Cardiac symptoms. **Table S3.** Confirmed diagnoses for those presenting with cardiac symptoms

## Data Availability

Aggregate data may be made available through a formal request to Hamad Medical Corporation, PO Box 3050, Doha, Qatar, who will make any and all decisions regarding data sharing. No personally identifiable data will be provided.

## Abbreviations

ED: Emergency Department
HGH: Hamad General Hospital
HH: Heart Hospital
