# Supplementary material for: Impact of COVID-19 upon changes in emergency room visits with chest pain of possible cardiac origin
Source: BMC Res Notes. 2020 Nov 18;13:539. doi: 10.1186/s13104-020-05381-y (PMC7672168; doi:10.1186/s13104-020-05381-y)
Supplement: Supplementary file 1 — Additional file 1: Table S1. Chief complaints of patients presenting to the Emergency Departments at all participating hospitals over the study period (listed alphabetically). Table S2. Cardiac symptoms. Table S3. Confirmed diagnoses for those presenting with cardiac symptoms [file 13104_2020_5381_MOESM1_ESM.docx]

Table S1. Chief complaints of patients presenting to the Emergency Departments at all participating hospitals over the study period (listed alphabetically).

|  | **2019-03** | **2019-04** | **2020-03** | **2020-04** | **Total** |
| --- | --- | --- | --- | --- | --- |
| Abdominal Pain/ Symptoms | 2,973 | 2,920 | 1,884 | 1,305 | 9,082 |
| Abnormal Behavior | 99 | 93 | 97 | 96 | 385 |
| Abnormal Test Result | 96 | 103 | 73 | 19 | 291 |
| Abscess | 381 | 378 | 180 | 142 | 1,081 |
| Alcohol Intoxication (Related) | 61 | 48 | 60 | 56 | 225 |
| Allergy or allergic Reaction | 307 | 298 | 198 | 107 | 910 |
| Altered Mental Status | 10 | 6 | 4 | 11 | 31 |
| Amputation | 8 | 20 | 7 | 11 | 46 |
| Animal Bite/ Scratch | 233 | 226 | 106 | 100 | 665 |
| Anxiety | 70 | 92 | 92 | 84 | 338 |
| Arrythmia | 16 | 16 | 16 | 19 | 67 |
| Assault | 263 | 256 | 181 | 172 | 872 |
| Asthma | 12 | 18 | 8 | 8 | 46 |
| Back Pain | 1,713 | 1,675 | 946 | 541 | 4,875 |
| Bleeding | 240 | 219 | 142 | 91 | 692 |
| Body Pain or Weakness | 29 | 35 | 41 | 33 | 138 |
| Breast Pain/ Lump/ Discharge/ Other | 79 | 84 | 49 | 38 | 250 |
| Burn | 123 | 128 | 63 | 51 | 365 |
| Cast Care | 7 | 14 | 3 | 8 | 32 |
| Chest Pain Likely Cardiac | 2,090 | 1,860 | 1,447 | 860 | 6,257 |
| Chest Pain/ Other | 148 | 132 | 118 | 83 | 481 |
| Chickenpox | 30 | 31 | 14 | 14 | 89 |
| Cough | 1,136 | 1,090 | 2,733 | 1,148 | 6,107 |
| Dental Issue | 50 | 45 | 33 | 12 | 140 |
| Dizziness | 935 | 940 | 664 | 375 | 2,914 |
| Dysuria/ Hematuria | 200 | 196 | 91 | 55 | 542 |
| Ear Pain/ Discharge/ Other | 420 | 470 | 202 | 106 | 1,198 |
| Edema/ Swelling | 239 | 221 | 132 | 88 | 680 |
| Epigastric Pain | 340 | 344 | 216 | 174 | 1,074 |
| Eye symptoms excluding foreign body | 1,220 | 1,232 | 655 | 289 | 3,396 |
| Fall | 703 | 684 | 417 | 382 | 2,186 |
| Fever | 2,069 | 1,946 | 3,488 | 3,523 | 11,026 |
| Foreign Body | 183 | 172 | 138 | 76 | 569 |
| Headache | 1,309 | 1,342 | 1,041 | 594 | 4,286 |
| High or low blood pressure | 292 | 219 | 194 | 125 | 830 |
| High or low blood sugar | 101 | 101 | 51 | 51 | 304 |
| Injury/ Trauma | 1,495 | 1,515 | 935 | 645 | 4,590 |
| Itching or rash | 302 | 332 | 152 | 66 | 852 |
| Missing | 1,804 | 1,744 | 2,005 | 3,218 | 8,771 |
| Nausea/ Vomiting/ Diarrhea (ANY) | 1,008 | 1,066 | 722 | 424 | 3,220 |
| Other | 19 | 24 | 15 | 16 | 74 |
| Pain/ Other | 4,505 | 4,276 | 2,479 | 1,417 | 12,677 |
| Palpitations | 336 | 317 | 273 | 144 | 1,070 |
| RTA | 436 | 391 | 316 | 198 | 1,341 |
| RTI | 1,019 | 822 | 1,587 | 589 | 4,017 |
| Shortness of Breath | 677 | 683 | 607 | 434 | 2,401 |
| SOB/ Cough or fever | 40 | 45 | 112 | 94 | 291 |
| Syncope/ Near Syncope/ Fainting | 82 | 81 | 62 | 34 | 259 |
| **Grand Total** | **29,908** | **28,950** | **25,049** | **18,126** | **102,033** |

Table S2. Cardiac symptoms.

|  | **2019-03** | **2019-04** | **2020-03** | **2020-04** | **Total** |
| --- | --- | --- | --- | --- | --- |
| Chest Pain Likely Cardiac | 2,090 | 1,860 | 1,447 | 860 | 6,257 |
| Palpitations | 336 | 317 | 273 | 144 | 1,070 |
| Shortness of Breath | 677 | 683 | 607 | 434 | 2,401 |
| Syncope/ Near Syncope/ Fainting | 82 | 81 | 62 | 34 | 259 |
| **Grand Total** | **3,185** | **2,941** | **2,389** | **1,472** | **9,987** |

Table S3. Confirmed diagnoses for those presenting with cardiac symptoms

|  | **2019-03** | **2019-04** | **2020-03** | **2020-04** | **Total** |
| --- | --- | --- | --- | --- | --- |
| Acute coronary syndrome | 191 | 167 | 170 | 113 | 641 |
| Allergic Reaction | 2 | 6 | 1 | 1 | 10 |
| Angina or coronary artery disease without acute coronary syndrome | 93 | 74 | 32 | 7 | 206 |
| Anxiety | 1 | 1 | 2 | 0 | 4 |
| Arrythmia | 78 | 42 | 47 | 26 | 193 |
| Asthma/ chronic obstructive pulmonary disease | 24 | 21 | 23 | 8 | 76 |
| Cardiac Arrest | 20 | 14 | 20 | 19 | 73 |
| Chest pain/ other or non-specific | 1,704 | 1,546 | 1,164 | 687 | 5,101 |
| Congestive heart failure | 62 | 47 | 63 | 35 | 207 |
| Cerebrovascular accident | 1 | 1 | 1 | 2 | 5 |
| Missing | 343 | 329 | 302 | 163 | 1,137 |
| Other | 136 | 145 | 91 | 50 | 422 |
| Respiratory tract infection | 26 | 28 | 25 | 12 | 91 |
| Shortness of breath/ Other | 503 | 513 | 443 | 348 | 1,807 |
| Transient ischemic attack | 0 | 1 | 0 | 0 | 1 |
| Valvular Disease | 1 | 6 | 5 | 1 | 13 |
| **Grand Total** | **3,185** | **2,941** | **2,389** | **1,472** | **9,987** |
